# Inhibition of Hepatitis C Virus in Mice by a Small Interfering RNA Targeting a Highly Conserved Sequence in Viral IRES Pseudoknot

**DOI:** 10.1371/journal.pone.0146710

**Published:** 2016-01-11

**Authors:** Jae-Su Moon, Seung-Hoon Lee, Eun-Jung Kim, Hee Cho, Wooseong Lee, Geon-Woo Kim, Hyun-Ji Park, Seung-Woo Cho, Choongho Lee, Jong-Won Oh

**Affiliations:** 1 Department of Biotechnology, Yonsei University, 50 Yonsei-ro, Seodaemun-gu, Seoul 120–749, Korea; 2 College of Pharmacy, Dongguk University, Goyang 410–820, Korea; University of British Columbia, CANADA

## Abstract

The hepatitis C virus (HCV) internal ribosome entry site (IRES) that directs cap-independent viral translation is a primary target for small interfering RNA (siRNA)-based HCV antiviral therapy. However, identification of potent siRNAs against HCV IRES by bioinformatics-based siRNA design is a challenging task given the complexity of HCV IRES secondary and tertiary structures and association with multiple proteins, which can also dynamically change the structure of this *cis*-acting RNA element. In this work, we utilized siRNA tiling approach whereby siRNAs were tiled with overlapping sequences that were shifted by one or two nucleotides over the HCV IRES stem-loop structures III and IV spanning nucleotides (nts) 277–343. Based on their antiviral activity, we mapped a druggable region (nts 313–343) where the targets of potent siRNAs were enriched. siIE22, which showed the greatest anti-HCV potency, targeted a highly conserved sequence across diverse HCV genotypes, locating within the IRES subdomain IIIf involved in pseudoknot formation. Stepwise target shifting toward the 5′ or 3′ direction by 1 or 2 nucleotides reduced the antiviral potency of siIE22, demonstrating the importance of siRNA accessibility to this highly structured and sequence-conserved region of HCV IRES for RNA interference. Nanoparticle-mediated systemic delivery of the stability-improved siIE22 derivative gs_PS1 siIE22, which contains a single phosphorothioate linkage on the guide strand, reduced the serum HCV genome titer by more than 4 log_10_ in a xenograft mouse model for HCV replication without generation of resistant variants. Our results provide a strategy for identifying potent siRNA species against a highly structured RNA target and offer a potential pan-HCV genotypic siRNA therapy that might be beneficial for patients resistant to current treatment regimens.

## Introduction

Hepatitis C virus (HCV) infection, which often evades innate and adaptive immune responses, leads to chronic liver diseases including cirrhosis and hepatocellular carcinoma. Therapeutic options for HCV patients have been expanded recently. Consequently, the previous standard therapy, the combination of pegylated interferon (PEG-IFN)-α and ribavirin, which is associated with serious side effects and assures a long term eradication of the virus in less than half of treated HCV patients [1], is being replaced by IFN-free regimens formulated with orally active, small molecule antiviral therapeutics. Several recently approved direct-acting antivirals (DAAs) including inhibitors targeting HCV nonstructural (NS) proteins such as NS3 protease, NS5B RNA-dependent RNA polymerase (RdRp), and NS5A protein improved clinical outcomes in patients with chronic hepatitis C. However, emergence of DAA-resistant viruses remains a challenge for sustained antiviral response (SVR) [2, 3]. Additionally, current DAAs did not provide satisfactory SVR against diverse HCV genotypes and subtypes [4, 5]. Therefore, there is a need for alternative pan-genotypic antiviral therapies with a high genetic barrier to resistance.

HCV is a positive-sense RNA virus and its 9.6-kb genome contains a single open reading frame (ORF) that encodes at least 10 viral proteins including various structural and NS proteins. The ORF is flanked by the 5′ and 3′ untranslated regions (UTRs), which contain conserved *cis*-acting RNAs essential for viral genome replication and translation and thus are attractive targets for designing nucleic acids-based anti-HCV agents [6–8]. The HCV 5′-UTR of 341 nucleotides (nts) in length contains an internal ribosomal entry site (IRES) that consists of four stem-loop (SL) structures followed by a translational initiation codon for the viral polypeptides [9, 10]. Among the four SL regions in HCV IRES, SLs II, III, and IV spanning nts 42–356 were shown to be essential for IRES activity [11, 12]. SL1 is not required for IRES function but the region located between SLI and SLII contain two important *cis*-acting sequences recognized by the liver abundant microRNA miR-122. Argonaute2 (Ago2)-dependent binding of miR-122 to these targets positively regulates HCV replication by multiple mechanisms [13–15]. The SLIII is also reported to be involved to some extent in genome replication [16]. Because of its important roles in translation and replication as well as its high sequence conservation across all HCV genotypes (seven genotypes and a series of subtypes), HCV IRES has been considered as a promising target for small interfering RNA (siRNA)-mediated antiviral therapy [17–19]. Although siRNA-based therapy is an attractive option, particularly for those HCV patients who are insensitive to the combination therapy with PEG-IFN-α and ribavirin, or who develop resistant variants against current DAAs, selection of potent siRNAs capable of accessing complicated secondary and tertiary structures within the sequence-conserved domains of HCV IRES is a challenging task. In addition, multiple proteins are recruited to IRES to stimulate translation and to enhance viral replication. These include so called IRES *trans*-acting factors (ITAFs) such as La protein, polypyrimidine tract-binding protein, poly(rC)-binding protein 2, and eukaryotic initiation factor 3, several heterogeneous nuclear RNA proteins [20] as well as 40S ribosomal subunit [8, 21] and miR-122 [13, 22]. Those factors can compete with siRNAs for binding to a common target to block the access of siRNAs. In addition, their interaction with HCV IRES can cause dynamic changes in its structure thereby masking siRNA-binding sites. Due to the above-described features of HCV IRES, sequence-based bioinformatics approaches have a limited use in identifying siRNAs that can effectively access the target in the milieu of multiple IRES-interacting host factors.

In the present study, we screened a set of siRNAs tiled over the HCV IRES SL structures III and IV and identified siIE22, a potent anti-HCV siRNA targeting a highly conserved sequence within the IRES subdomain IIIf, which participates in the formation of the pseudoknot 1 through base-pairing with the downstream sequence in the SL IV [23, 24]. We also investigated *in vivo* antiviral efficacy of a chemically modified siIE22 derivative, which contain a single phosphorothioate linkage on the guide strand, in a mouse model of HCV replication. We report the clinical application potential of the IRES-targeting siRNA therapy for the treatment of HCV infection.

## Materials and Methods

### Plasmids, antibodies, and reagents

The JFH1 plasmid [25] was used to produce infectious HCV particles. The pRluc-JFH1 plasmid [26] harboring *Renilla* luciferase (Rluc)-coding gene in the context of genotype 2a full-length HCV JFH1 cDNA clone was received from Dr. Xulin Chen (Institute for Virus Research, Chinese Academic of Sciences, Wuhan, China). The Rluc-J6/JFH1 (FL-J6/JFH-5′C19Rluc2AUbi) plasmid [27], which was derived from the previously described infectious genotype 2a chimeric HCV genome J6/JFH1 [28], was used for the synthesis of a full-length HCV genome that expresses Rluc. The bicistronic vector pDual-IRES, which expresses a cap-dependent Rluc reporter and an HCV IRES-dependent firefly luciferase (Fluc) reporter was described previously [29]. The pcDNA3.1-Ago2 vector expressing the human Ago2 was constructed by RT-PCR-mediated amplification of Ago2 cDNA using the forward primer Ago2-EcoRI-F (5′-gactGAATTCgATGTACTCGGGAGCCGGCCCCGCACT-3′) and the reverse primer Ago2-NotI-R (5′-gactGCGGCCGCTCAAGCAAAGTACATGGTG-3′), followed by cloning of the PCR-amplified product into the *Eco*RI and *Not*I-cleaved pcDNA3.1 vector (Invitrogen, Carlsbad, CA, USA). Recombinant human Ago2 was obtained from Sino Biological Inc. (Beijing, China). The antibody against HCV NS5A was obtained from Biodesign International (clone 381; Kennebunk, ME, USA). The mouse anti-α-tubulin antibody was from Calbiochem (La Jolla, CA, USA). The rabbit polyclonal anti-NS5B antibody was generated previously in our laboratory [30]. The goat anti-HCV E2 polyclonal antibody was obtained from Acris Antibodies (GmbH, Hideenhausen, Germany). Polyinosinic:polycytidylic acid [poly(I:C)], a double-stranded RNA (dsRNA) analog, was obtained from Sigma-Aldrich (St. Louis, MO, USA).

### Cell culture

The Huh7 human hepatocellular carcinoma and human embryonic kidney 293 (HEK293) cell lines were grown in the Dulbecco’s modified Eagle’s medium (DMEM) as reported previously [31]. The R-1 cell line, in which a selectable HCV subgenomic replicon replicates [32], was also maintained in DMEM supplemented with 1 mg/ml of G418. Human peripheral mononuclear cells (hPBMCs) were isolated by centrifugation over Ficoll-Hypaque (GE Healthcare, Uppsala, Sweden) and grown as previously described [31].

### siRNA and transfection

The siRNAs targeting HCV IRES were obtained from ST Pharm (Seoul, Korea). The sequences of siRNAs used in this study are shown in Table 1. Several chemically modified siIE22 derivatives were synthesized and their sequences are shown in the corresponding figure in the results section. In one set of siIE22 derivatives, the 2′-OH group in the ribose sugar backbone was substituted with the 2′-O-methyl (2′O-Me) or 2′-fluoro (2′F) groups, while another derivative carried a replacement of one of the non-bridging oxygen atom from the backbone phosphate between two ribonucleotides with a sulfur atom to create a phosphorothioate (PS) linkage. siRNAs were transfected into cells using Lipofectamine RNAiMAX (Invitrogen) or the lipidoid ND98 as previously reported [33].

**Table 1:** siRNAs used in this study.

siRNA	Sequence ^a^	Position (nt) ^b^
siIE1	5′-GGC CUU GUG GUA CUG CCU G**UU**-3′	277–295
	3′-**UU** CCG GAA CAC CAU GAC GGA C-5′	
siIE2	5′-CCU UGU GGU ACU GCC UGA U**UU**-3′	279–297
	3′-**UU** GGA ACA CCA UGA CGG ACU A-5′	
siIE3	5′-UUG UGG UAC UGC CUG AUA G**UU**-3′	281–299
	3′-**UU** AAC ACC AUG ACG GAC UAU C-5′	
siIE4	5′-GUG GUA CUG CCU GAU AGG G**UU**-3′	283–301
	3′-**UU** CAC CAU GAC GGA CUA UCC C-5′	
siIE5	5′-GGU ACU GCC UGA UAG GGU G**UU**-3′	285–303
	3′-**UU** CCA UGA CGG ACU AUC CCA C-5′	
siIE6	5′-UAC UGC CUG AUA GGG UGC U**UU**-3′	287–305
	3′-**UU** AUG ACG GAC UAU CCC ACG A-5′	
siIE7	5′-CUG CCU GAU AGG GUG CUU G**UU**-3′	289–307
	3′-**UU** GAC GGA CUA UCC CAC GAA C-5′	
siIE8	5′-GCC UGA UAG GGU GCU UGC G**UU**-3′	291–309
	3′-**UU** CGG ACU AUC CCA CGA ACG C-5′	
siIE9	5′-CUG AUA GGG UGC UUG CGA G**UU**-3′	293–311
	3′-**UU** GAC UAU CCC ACG AAC GCU C-5′	
siIE10	5′-GAU AGG GUG CUU GCG AGU G**UU**-3′	295–313
	3′-**UU** CUA UCC CAC GAA CGC UCA C-5′	
siIE11	5′-UAG GGU GCU UGC GAG UGC C**UU**-3′	297–315
	3′-**UU** AUC CCA CGA ACG CUC ACG G-5′	
siIE12	5′-GGG UGC UUG CGA GUG CCC C**UU**-3′	299–317
	3′-**UU** CCC ACG AAC GCU CAC GGG G-5′	
siIE13	5′-GUG CUU GCG AGU GCC CCG G**UU**-3′	301–319
	3′-**UU** CAC GAA CGC UCA CGG GGC C-5′	
siIE14	5′-GCU UGC GAG UGC CCC GGG A**UU**-3′	303–321
	3′-**UU** CGA ACG CUC ACG GGG CCC U-5′	
siIE15	5′-UUG CGA GUG CCC CGG GAG G**UU**-3′	305–323
	3′-**UU** AAC GCU CAC GGG GCC CUC C-5′	
siIE16	5′-GCG AGU GCC CCG GGA GGU C**UU**-3′	307–325
	3′-**UU** CGC UCA CGG GGC CCU CCA G-5′	
siIE17	5′-GAG UGC CCC GGG AGG UCU C**UU**-3′	309–327
	3′-**UU** CUC ACG GGG CCC UCC AGA G-5′	
siIE18	5′-GUG CCC CGG GAG GUC UCG U**UU**-3′	311–329
	3′-**UU** CAC GGG GCC CUC CAG AGC A-5′	
siIE19	5′-GCC CCG GGA GGU CUC GUA G**UU**-3′	313–331
	3′-**UU** CGG GGC CCU CCA GAG CAU C-5′	
siIE21	5′-CGG GAG GUC UCG UAG ACC G**UU**-3′	317–335
	3′-**UU** GCC CUC CAG AGC AUC UGG C-5′	
siIE21.5	5′-GGG AGG UCU CGU AGA CCG U**UU**-3′	318–336
	3′-**UU** CCC UCC AGA GCA UCU GGC A-5′	
siIE22	5′-GGA GGU CUC GUA GAC CGU G**UU**-3′	319–337
	3′-**UU** CCU CCA GAG CAU CUG GCA C-5′	
siIE22.5	5′-GAG GUC UCG UAG ACC GUG C**UU**-3′	320–338
	3′-**UU** CUC CAG AGC AUC UGG CAC G-5′	
siIE23	5′-AGG UCU CGU AGA CCG UGC A**UU**-3′	321–339
	3′-**UU** UCC AGA GCA UCU GGC ACG U-5′	
siRNA	Sequence	Position (nt)
siIE24	5′-GUC UCG UAG ACC GUG CAC C**UU**-3′	323–341
	3′-**UU** CAG AGC AUC UGG CAC GUG G-5′	
siIE25	5′-CUC GUA GAC CGU GCA CCA U**UU**-3′	325–343
	3′-**UU** GAG CAU CUG GCA CGU GGU A-5′	
si313	5′-CCC GGG AGG UCU CGU AGA C**TT**-3′	315–333
	3′-**TT** GGG CCC UCC AGA GCA UCU G-5′	
siE	5′-GUC UCG UAG ACC GUG CAU CA **UU**-3′	323–342
	3′-**UU** CAG AGC AUC UGG CAC GUA GU-5′	
siIE318_27	5′-GGG AGG UCU CGU AGA CCG UGC ACC A **UU**-3′	318–342
	3′-**UU** CCC UCC AGA GCA UCU GGC ACG UGG U-5′	
siIE320_25	5′-GAG GUC UCG UAG ACC GUG CAC CA **UU**-3′	320–342
	3′-**UU** CUC CAG AGC AUC UGG CAC GUG GU-5′	
siIE322_23	5′-GGU CUC GUA GAC CGU GCA CCA **UU**-3′	322–342
	3′-**UU** CCA GAG CAU CUG GCA CGU GGU-5′	
siIE316	5′-CCG GGA GGU CUC GUA GAC C**dTdT**-3′	316–334
	3′-**dTdT** GGC CCU CCA GAG CAU CUG G-5′	
siIE322	5′-GGU CUC GUA GAC CGU GCA C**dTdT**-3′	322–340
	3′-**dTdT** CCA GAG CAU CUG GCA CGU G-5′	
siIE319	5′-GGA GGU CUC GUA GAC CGU G**CA**-3′	319–339
	3′-**GC** CCU CCA GAG CAU CUG GCA C-5′	

^a^ Underlined and boldface letters indicate 3' overhang sequence added to the 3'-ends of siRNAs.

^b^ siRNA target positions are numbered according to the HCV replicon Con1 sequence (genotype 1b; GenBank accession number AJ238799).

### LNP formulation and nanoparticle size analysis

For systemic siRNA delivery, siRNA LNPs were prepared using ND98, cholesterol (Sigma-Aldrich), and polyethylene glycol (PEG) 2000-ceramide C16 (Avanti Polar Lipids, Alabaster, AL, USA), as previously described [33]. The concentration of siRNA encapsulated within LNPs was determined using the Quant-iT RiboGreen RNA assay [33]. LNP particle size was analyzed using a NanoSight LM10 instrument (NanoSight, Amesbury, UK) according to the manufacturer’s instructions. Briefly, a monochromatic laser beam at 405 nm was applied to PBS-diluted (10,000-fold dilution) siIE22 LNPs. Brownian motion of LNPs was tracked and measured from frame to frame using nanoparticle tracking analysis software. The velocity of moving particles was used to calculate particle size from the 2D Stokes–Einstein equation.

### Target RNA cleavage assays

An *in vitro* RNA cleavage assay was performed using a human recombinant Ago2 as described previously [34]. Alternatively, the human Ago2 immunoprecipitated from pcDNA3.1-Ago2-transfected HeLa cells was also used as previously reported [35]. Briefly, Ago2, which forms an active RNA-induced silencing complex (RISC) complex in cells, was immunoprecipitated using a rabbit anti-Ago2 antibody (clone C34C6; Cell Signaling Technology, Danvers, MA, USA) and protein G Dynabeads (Dynal, Oslo, Norway). Target RNAs used for the Ago2 cleavage assays included HCV genome 5′-end region (488-nts including an extra G residue added to the 5′-end) and an HCV IRES region spanning nts 313–343 that were 5′-end ^32^P-labeled using [γ-^32^P] ATP and T4 polynucleotide kinase. RNA samples were resolved on a denaturing polyacrylamide gel for autoradiography.

### HCV infection

Full-length HCV (genotype 2a, JFH1 clone) RNA was prepared by *in vitro* transcription and electroporated into Huh7 cells according to the protocol reported previously [36]. Infectious HCV particles collected from the culture medium were used for infection of Huh7 cells as previously described [31].

### Transient HCV replication assay

Huh7 cells were co-transfected by electroporation with *in vitro* transcripts of pRluc-JFH1 and pGL3 plasmids (Promega, Madison, WI, USA). After 24 h, siRNAs were transfected using Lipofectamine RNAiMAX (Invitrogen). Rluc and Fluc activities in cell lysates were monitored 48 h after transfection using the Dual-Glo luciferase assay system (Promega). Rluc activity was normalized against Fluc activity to calculate relative luciferase activity.

### TaqMan real-time quantitative RT-PCR

The HCV genome copy number was estimated by real-time quantitative RT-PCR (qRT-PCR) using a TaqMan probe specific for the HCV 5′-UTR as previously described [29].

### Western blot and northern blot analyses

Western blot analysis of HCV viral proteins was performed as described previously [37]. For HCV genome detection by northern blotting, total RNA (5 μg) run on a denaturing formaldehyde agarose gel was blotted and hybridized with a ^32^P-labeled DNA probe [HCV NS3 NS5B (nts 3446–9265)] prepared by using the Ready-To-Go DNA labeling kit (GE Healthcare Life Sciences, Piscataway, NJ, USA).

### Interferon β promoter activity assay

HEK293 cells grown in 24-well plates were transfected with 400 ng of IFNβ-pGL3, which expresses Fluc under the control of the IFN-β promoter [38] and 100 ng of the pRL-TK reporter (Promega), which expresses Rluc as an internal control. Six hours after transfection, cells were washed, incubated with fresh medium, and transfected with siRNA (100 nM) or poly(I:C) (1 μg/ml) using Lipofectamine RNAiMAX (Invitrogen). Stimulated cells were harvested 8 h later, and luciferase assays were performed using the Dual-Glo luciferase assay system (Promega) as described above.

### Cell viability

The cytotoxicity of siIE22 was measured using MTS [3-(4,5-dimethylthiazol-2-yl)-2,5-diphenyltetrazolium bromide] reagent as previously described [31].

### siRNA stability test

Serum stability of siRNAs was assessed by northern blot analysis. siRNA (2 μM) was incubated in 45% human plasma at 37°C for indicated times prior to northern blot analysis by a conventional method. Briefly, total RNA extracted with Trizol LS reagent (Invitrogen) was separated by 15% polyacrylamide/7 M urea gel electrophoresis and transferred to a membrane by electroblotting. As a probe, 5′-end ^32^P-labeled, synthetic ribo-oligonucleotide complementary to siRNA guide-strand was used. Densitometric quantification of the radioactive hybridization signal was performed using a Fuji BAS-2500 Phosphorimager.

### Enzyme-linked immunosorbent assay

The experiment with hPBMCs was approved by the Institutional Review Board of the Yonsei University (Approval No: 1040917-201312-BR-133-01E). hPBMCs were isolated from peripheral blood, which was obtained from healthy volunteers with their written informed consent and provided by the Korean Red Cross, Western Blood Center Blood Services (Approval No: 2823–201312). hPBMCs (1 × 10^6^ cells/well) grown in a 96-well round-bottom plate were transfected with siRNA (10 nM) or poly(I:C) (1 μg/well) using ND98 or LNP. As a positive control for these assays, 50 μg/ml of poly(I:C) was added directly to the culture medium. After stimulation for 16 h, stimulated cultured cells were centrifuged, supernatants were collected by centrifugation, and the amount of IFN-α was determined using a human IFN-α⋅enzyme-linked immunosorbent assay (ELISA) kit (eBioscience, San Diego, CA, USA). The detection limit of the human IFN-α ELISA was 15 pg/ml. Serum IFN-α⋅titer in BALB/c mice (n = 4 per group) that received siRNA or poly(I:C) (1 mg RNA/kg body weight) either complexed with ND98 or formulated in LNPs was determined by ELISA (detection limit: 12.5 pg/ml) using a kit supplied by PBL Interferon Source (Piscataway, NJ, USA).

### Immunohistochemistry

Xenograft tissues fixed in 4% paraformaldehyde in PBS were embedded in a frozen section compound (Surgipath FSC22, Leica Microsystems, Wetzlar, Germany). Tissue sections were incubated for 2 h at room temperature with an affinity-purified polyclonal anti-NS5B antibody or a goat anti-HCV E2 polyclonal antibody with gentle rocking. After washing 3 times with PBS, appropriate fluorescent-conjugated secondary antibodies were used for antigen visualization. Nuclei were visualized by staining with 1 μM 4′,6′-diaamidino-2-phenylindole (DAPI) in PBS for 10 min. Confocal images were collected on an LSM 510 META confocal laser scanning microscope (Carl Zeiss, Oberkochen, Germany).

### Animal experiments

All animal experiments were performed in accordance with the Korean Food and Drug Administration guidelines. Protocols were reviewed and approved by the Institutional Animal Care and Use Committee of the Yonsei Laboratory Animal Research Center (Permit No: 2012–0076). At the termination of experiments, all mice were euthanized by CO_2_ inhalation.

### *In vivo* RNAi assay

The dual luciferase expression vector pDual-IRES (20 μg in 1.6 ml PBS), which was purified with the Endotoxin Free Maxi Kit (Qiagen, Hilden, Germany), was administered to 6–8 weeks old female BALB/c mice by hydrodynamic injection through the tail vein within 5 to 8 s. After 1 h, mice were intravenously (iv) injected with siRNA LNPs at a dose of 1 mg siRNA per kg body weight. After 16 h, livers were homogenized and mixed with an equal volume of the Glo lysis 1× buffer (Promega). Then, the mixture was centrifuged for 5 min at 13,000 *g* at 4°C to obtain cleared lysate that was analyzed for reporter activity using the Dual-Glo luciferase assay system (Promega). Luciferase expression was reported as relative light units (RLUs) per mg protein.

### Evaluation of *in vivo* siRNA anti-HCV activity

To evaluate *in vivo* anti-HCV efficacy of siRNA, NOD-SCID mice bearing HCV-replicating Huh7 xenograft were used. HCV RNA-transfected cells mixed with Matrigel (BD Biosciences Discovery Labware, Bedford, MA, USA) were injected subcutaneously (sc) into both flanks of anesthetized immunodeficient NOD-SCID male mice (5 weeks of age, 20–23 g body weight). siIE22 LNPs (1 mg siRNA per kg body weight) were administered by tail vein injection at 3 to 4 weeks after implantation.

### Statistical analysis

Statistical analysis was performed using GraphPad Prism (Graphpad Prism Software Inc., La Jolla, CA, USA). Results are presented as the mean ±standard deviation (SD) from at least three independent experiments, unless otherwise stated. Differences between groups were considered statistically significant if *P* < 0.05.

## Results

### Identification of potent HCV IRES-targeting siRNAs by siRNA-tiling experiments

**Fig 1:**
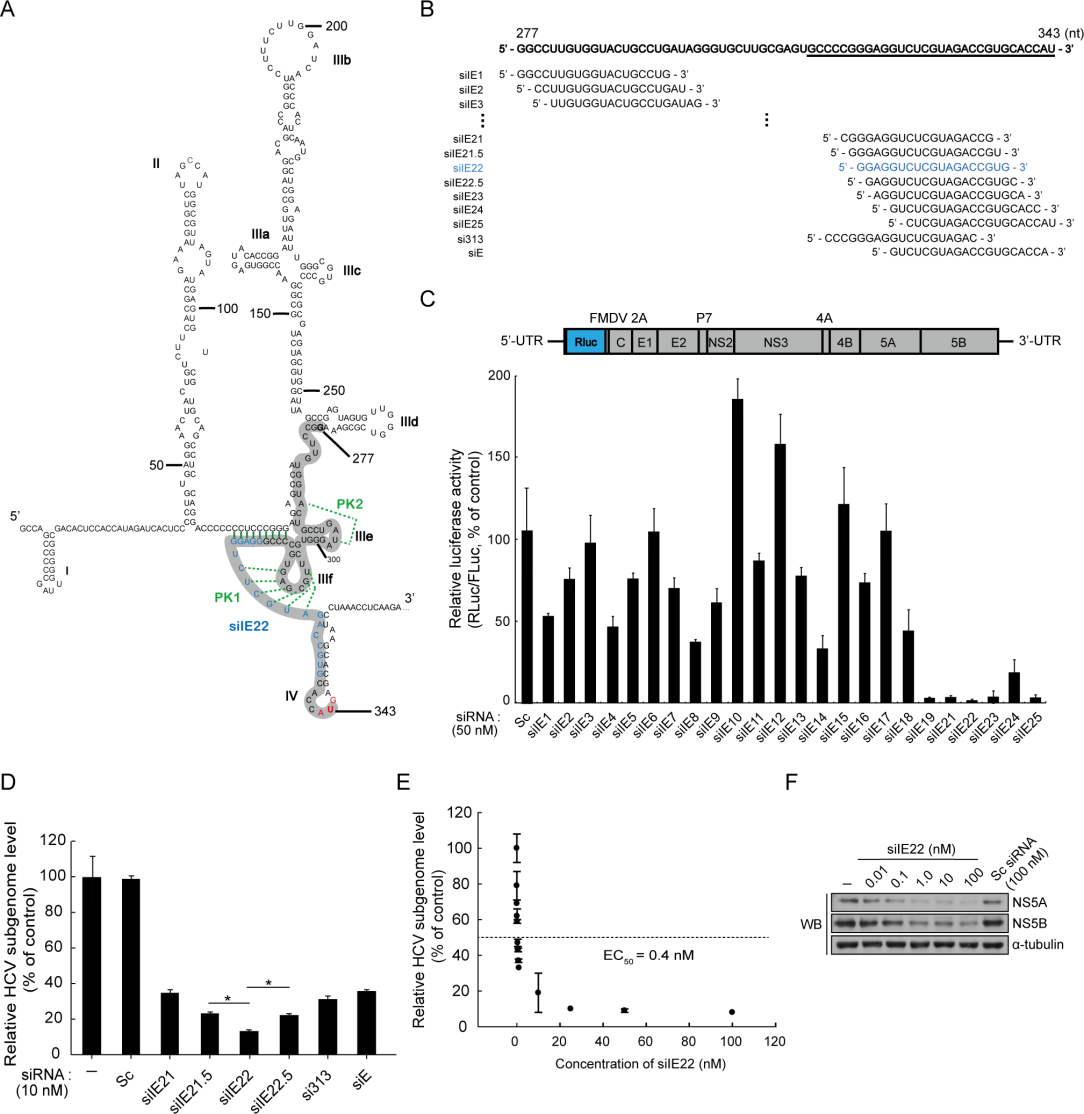
Screening for potent HCV IRES-targeting siRNA by siRNA tiling experiments. (A) The proposed secondary structure of HCV IRES. The IRES region spanning nts 277–343 (shown in gray) was targeted by siRNAs. The target site of the selected potent anti-HCV siRNA siIE22 is shown in blue. The base-pairings in the proposed pseudoknot (PK) structures (PKs 1 and 2) are shown in green. (B) siRNA sequences tiled over HCV IRES. The underlined sequence represents a mapped druggable region (nts 313–343) where the targets of selected potent siRNAs were enriched. (C) Anti-HCV activity of HCV IRES-targeting siRNAs in Huh7 cells transfected with Rluc-JFH1 (top panel), an HCV replicon encoding the Rluc reporter. The Rluc gene was fused in frame to the DNA sequence encoding 17 N-terminal amino acid residues of the HCV core protein. Huh7 cells were electroporated with the Rluc-JFH1 *in vitro* RNA transcript and pGL3 plasmid used for normalization of transfection efficiency. After 24 h, the cells were transfected with each of the IRES-specific siRNAs or a scrambled (Sc) siRNA (50 nM). At 48 h post-transfection, luciferase activity was measured. (D) Huh7 cells harboring an HCV subgenomic replicon RNA (R-1) were transfected with each of the IRES-specific siRNAs or Sc siRNA (10 nM each). At 48 h post-transfection, HCV RNA levels were quantified by real-time qRT-PCR. *, *P <* 0.01. (E and F) Dose-dependent inhibition of HCV-replication by siIE22 was assessed in R-1 cells, as described in (D). HCV genome copy number and HCV proteins (NS5A and NS5B) levels were analyzed by qRT-PCR (E) and western blotting (F), respectively.

To identify siRNA-targetable regions within highly structured HCV IRES, which is also associated with multiple cellular proteins, we utilized siRNA tiling approach as follows. A total of 25 siRNAs were initially tiled with overlapping sequences that were shifted by two nucleotides over the IRES SL region spanning nts 277–343 (Fig 1A, siRNA tiled region is shown in thick gray line; see also siRNA sequences in Table 1 and Fig 1B). We assessed anti-HCV activity of synthetic siRNAs in Huh7 cells transiently transfected with *in vitro* transcripts of the full-length genotype 2a HCV replicon Rluc-JFH1 encoding an Rluc reporter and identified six highly potent anti-HCV siRNAs (siIE19, siIE21, siIE22, siIE23, siIE24, and siIE25) targeting the SL IIIf and IV regions spanning nts 313–343 (Fig 1C). Other siRNAs targeting sites upstream of these IRES regions showed less potent antiviral activity than the selected siRNAs. RNAi effect of non-selected siRNAs was marginal and sometimes application of these siRNAs had an unexpected boosting effect on virus replication. Among the selected antiviral siRNAs, siIE22 targeting nts 319–337 was identified as the most effective siRNA, reducing the reporter activity by up to 98% at a 50 nM concentration.

Our results described above underscore the importance of siRNA targeting site selection within the highly structured IRES in executing RNAi. To further support this conclusion, we designed two additional siRNAs—siIE21.5 and siIE22.5—targeting the regions around the siIE22 binding site (1-nt shifting toward the 5′ and 3′ direction, respectively; see Fig 1B). For this purpose, we also included two other previously reported potent anti-HCV siRNAs—si313 [39] and siE [40]—for antiviral efficacy assays because the si313 target (nts 315–333) partially overlaps with binding sites of siIE19 (nts 313–331) and siIE21 (nts 317–335). siE, which is 1 nt longer than siIE24 (targeting nts 323–341), targets nts 323–342. We found that siIE22 displayed greater antiviral potency in R-1 cells harboring a genotype 1b HCV subgenomic replicon (Fig 1D), although the siIE22 target sequence overlaps with the target sites of other tested siRNAs described above. Importantly, we observed that siIE21.5 and siIE21 showed lower antiviral activity than siIE22, as their targets were shifted by 1 nt and 2 nts toward the 5′-direction, respectively. Similarly, sequential nucleotide shifting of the siIE22 target site toward the 3′-direction also attenuated the antiviral potency of siIE22 (compare siIE22 with siIE22.5, siIE23, siIE24, siE, and siIE25 in Fig 1C and 1D). These results clearly map a short 31-nt stretch ranging from nt 313 to nt 343 as the druggable target site for potent anti-HCV siRNAs. siIE22 targeted a previously unidentified 19-nts sequence array from nt 319 to nt 337 within the IRES subdomain IIIf (Fig 1A) involved in the formation of the pseudoknot 1 [24]. It showed the greatest anti-HCV potency with a half-maximum effective concentration (EC_50_) of 0.4 nM in R-1 cells (Fig 1E). Expression of HCV NS5A and NS5B proteins, as assessed by western blot analysis, was decreased dose-dependently by the treatment with siIE22 (Fig 1F). Results of siRNA tiling experiments showed that even a single nucleotide target shift toward either 5′ or 3′ direction could lower the antiviral potency of siIE22 suggesting a specific siRNA positioning effect.

**Fig 2:**
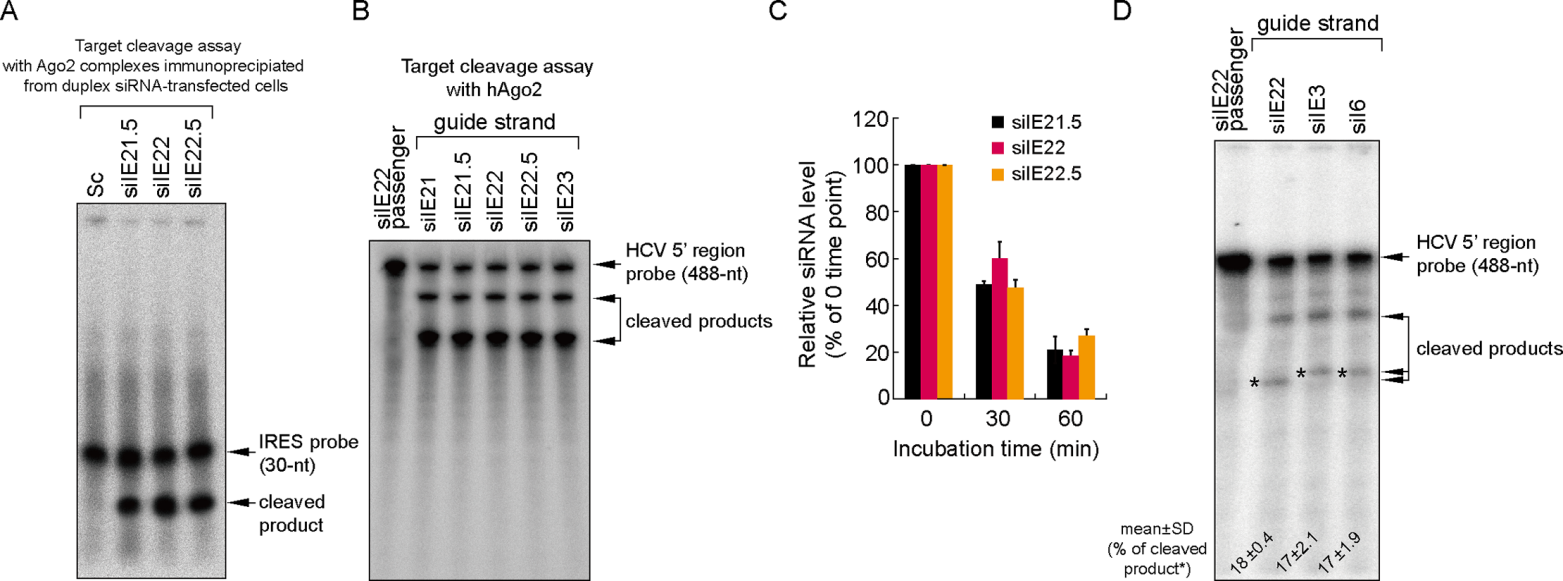
Target RNA cleavage activity and serum stability of the selected IRES-targeting siRNAs. (A) *In vitro* cleavage assays were performed with 5′ ^32^P-radiolabeled 31-nts long HCV IRES (spanning nts 313–343) using Ago2 complexes immunoprecipitated from HeLa cells transfected with each indicated siRNA (20 nM). Sc, scrambled siRNA. (B) RNA target cleavage assay was performed with 5′ ^32^P-radiolabeled HCV IRES substrate (spanning nts 1–487) using a recombinant human Ago2 protein and guide strand of indicated siRNAs. As a negative control, siIE22 passenger-strand was used. RNA samples were analyzed by electrophoresis on a denaturing polyacrylamide denaturing gel followed by autoradiography. (C) Serum stability of siRNAs was assessed by incubating siRNAs in 45% plasma for the indicated time periods and subsequent quantification of remained intact siRNA guide-strand by Phosphorimager analysis of northern blots. (D) The cleavage assay was performed as described in (B). Radioactivity of cleaved products was quantified by Phosphorimager analysis and shown below the autoradiogram (mean ± SD of three independent experiments).

We reasoned that this superior antiviral activity of siIE22 could be mediated by a greater target cleavage activity. To test this possibility, we performed IRES-target cleavage assays using immoprecipitated Ago2 (Fig 2A), which forms an active RISC in cells treated with a duplex form of siRNA [35], and using a recombinant Ago2 protein (Fig 2B), which uses single-stranded antisense siRNA guide-strand for RNAi [34]. We observed that two siRNAs siIE21.5 and siIE22.5, whose targets were shifted by a single nucleotide toward the 5′ and 3′ directions, respectively, relative to the siIE22 target site, as well as siRNAs siIE21 and siIE23, whose targets were similarly shifted by two nucleotides, were capable of digesting the probe equally well *in vitro*. Subsequent analysis of the stability of siIE21.5 and siIE22.5 in human plasma showed no noticeable differences in siRNA stability between these siRNAs and siIE22 over the period of 60 min incubation at 37°C (Fig 2C). Considering the fact that HCV IRES is bound by various ITAFs as well as by ribosomal complexes [8, 21], these results collectively suggest that the strong antiviral activity of siIE22 might be due to its optimal target accessibility in cellular context. Further supporting this conclusion, although the two siRNAs targeting the pseudoknot 2 [24] (Fig 1A), siIE3 and siIE6, showed only negligible antiviral activity (Fig 1C), both of them were capable of cleaving the target probe as effectively as siIE22 (Fig 2D).

### Both the sequence and the length of siRNA are important in designing potent siRNAs targeting the IRES subdomain IIIf within the pseudoknot 1

**Fig 3:**
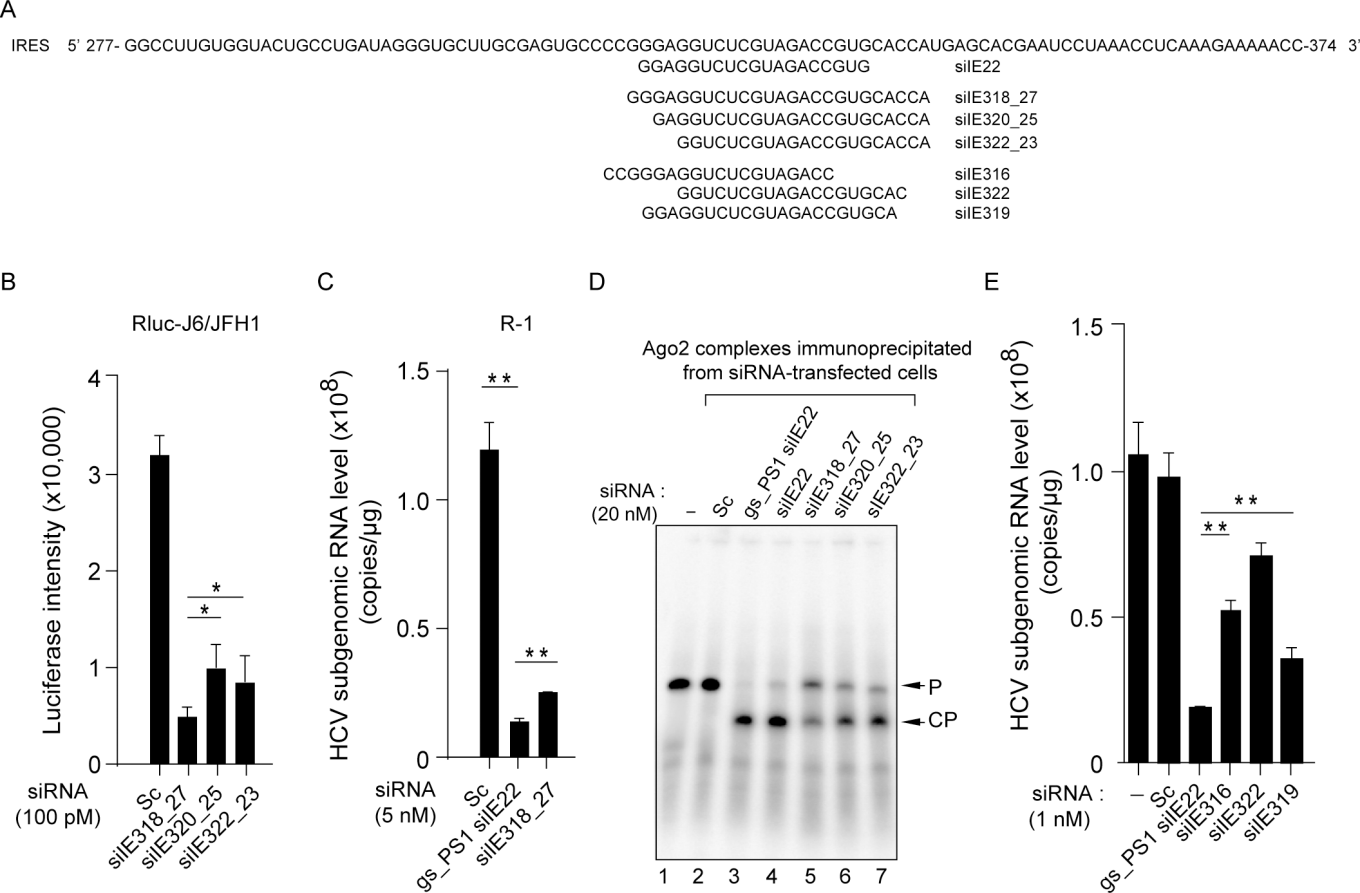
Analysis of anti-HCV potency and RNAi activity of various siRNAs targeting the IRES subdomain IIIf. (A) Passenger strand sequences of siIE22 and six siRNAs sharing their targets with siIE22 in the IRES subdomain IIIf. (B) Evaluation of anti-HCV efficacy of a set of selected siRNAs (100 pM each) using an HCV replicon expressing an Rluc reporter. Luciferase activity at 48 h post-siRNA treatment is shown. Sc, scrambled siRNA. (C) Antiviral efficacy of gs_PS1 siIE22 and siIE318_27 in R-1 cells harboring an HCV subgenomic replicon. (D) *In vitro* target cleavage assays were performed as described in Fig 2A. P, 5′ ^32^P-radiolabeled 31-nt long HCV IRES probe; CP, cleaved probe. (E) Comparison of antiviral activity of indicated siRNAs targeting the region shared with siIE22 in R-1 cells. In (B), (C), and (E), *, *P <* 0.05; **, *P <* 0.01.

We designed three additional synthetic siRNAs—siIE318_27 (27-mer; targeting nts 318–342), siIE320_25 (25-mer; targeting nts 320–342), and siIE322_23 (23-mer; targeting nts 322–342) (Fig 3A; also see their sequences in Table 1)—to test if these siRNAs, which contained the seed sequence of siIE22 (5′ 2–8 nts on the guide strand) but differed in the total length, display antiviral potency similar to that of siIE22. Of these three siRNAs, siIE318_27 showed the greatest antiviral activity with 85% inhibition at 100 pM in a cell line bearing a full-length HCV replicon encoding Rluc reporter (Fig 3B). However, siIE318_27 siRNA showed a lesser effect against the HCV replicon in R-1 cells when compared with gs_PS1 siIE22 (Fig 3C). Moreover, 27-mer siIE318_27 could not digest the IRES target as effectively as 21-mer siIE22 and its stabilized derivative gs_PS1 siIE22 (Fig 3D; compare lanes 3 and 4 with lane 5). The target cleavage assay also revealed that siIE320_25 and siIE322_23 displayed higher cleavage effects than siIE318_27 (Fig 3D, lanes 5–7). These cleavage assay results are consistent with an earlier report showing that siRNAs composed of more than 23 nts are less efficient in RNAi than 21-nt long siRNAs [41]. In this regard, we did not expect to observe that 27-mer siIE318_27 siRNA had a greater antiviral potency than the two smaller size siRNAs, siIE320_25 and siIE322_23. Considering that these siRNAs, as well as siE, all have the same 3**′** -end boundary (ending at the nt 342) in their targets with variable 5**′** -end boundary sequences (starting at the nt 318, 320, 322, and 323 for siIE318_27, siIE320_25 and siIE322_23, and siE, respectively), it appears that defining a specific binding site within the 31-nt druggable region (nts 313–343) mapped in the present study is critical for the identification of potent anti-HCV siRNAs.

Further, we tested another three 21-mer siRNAs (Table 1; they are all 21-mer siRNAs with a 3**′**-end two nucleotide overhang, including siIE316, siIE322, and siIE319) that share parts of their target site with siIE22 in antiviral activity comparison experiments. Of note, siIE319 shares 19 nts in its target sequence with siIE22; however, it differs from siIE22 in that the overhang sequences on both guide and passenger strands of siIE319 are derived from the HCV sequence and thus do not have TT or UU residues, the two conventional siRNA overhang sequences. Surprisingly, this difference resulted in a significant 1.78-fold reduction (*P* < 0.01) in the anti-HCV potency of siIE319 (Fig 3E), demonstrating the important role of overhang sequences in the formation of an active RISC complex with the guide strand chosen based on thermodynamic stability of siRNA termini. During the RNAi catalytic cycle, there is consecutive guide-strand 3**′**-end binding to and release from the PAZ domain of Ago2 [42]. It is thus likely that unfavorable interactions between 3**′**-end of the guide strand (GC in siIE319 vs. UU in siIE22) with the PAZ domain of Ago2 might interfere with active RISC formation and result in weaker RNAi. This hypothesis is supported by recent findings of Kandeel and Kitade [43], who demonstrated that weaker binding of siRNAs by the PAZ domain correlates with higher RNAi.

It should be noted that the siIE316 target is shifted by 1 nt toward the 5**′**-direction relative to siIE21 and that the siIE322 target is shifted by 1 nt toward the 3**′**-direction relative to siIE23. None of these siRNAs showed a stronger inhibitory effect than siIE22. A shift by three nts of the siIE22 target toward the 5**′**-direction to reach the 5**′** boundary of the siIE316 target reduced antiviral potency. Together, these results further highlight the importance of defining 5**′**- and 3**′**-ends target boundaries within the mapped siRNA accessible region in the HCV IRES subdomain IIIf for the identification of potent therapeutic siRNAs.

### *In vivo* inhibition of HCV IRES-mediated translation by siIE22 LNP

**Fig 4:**
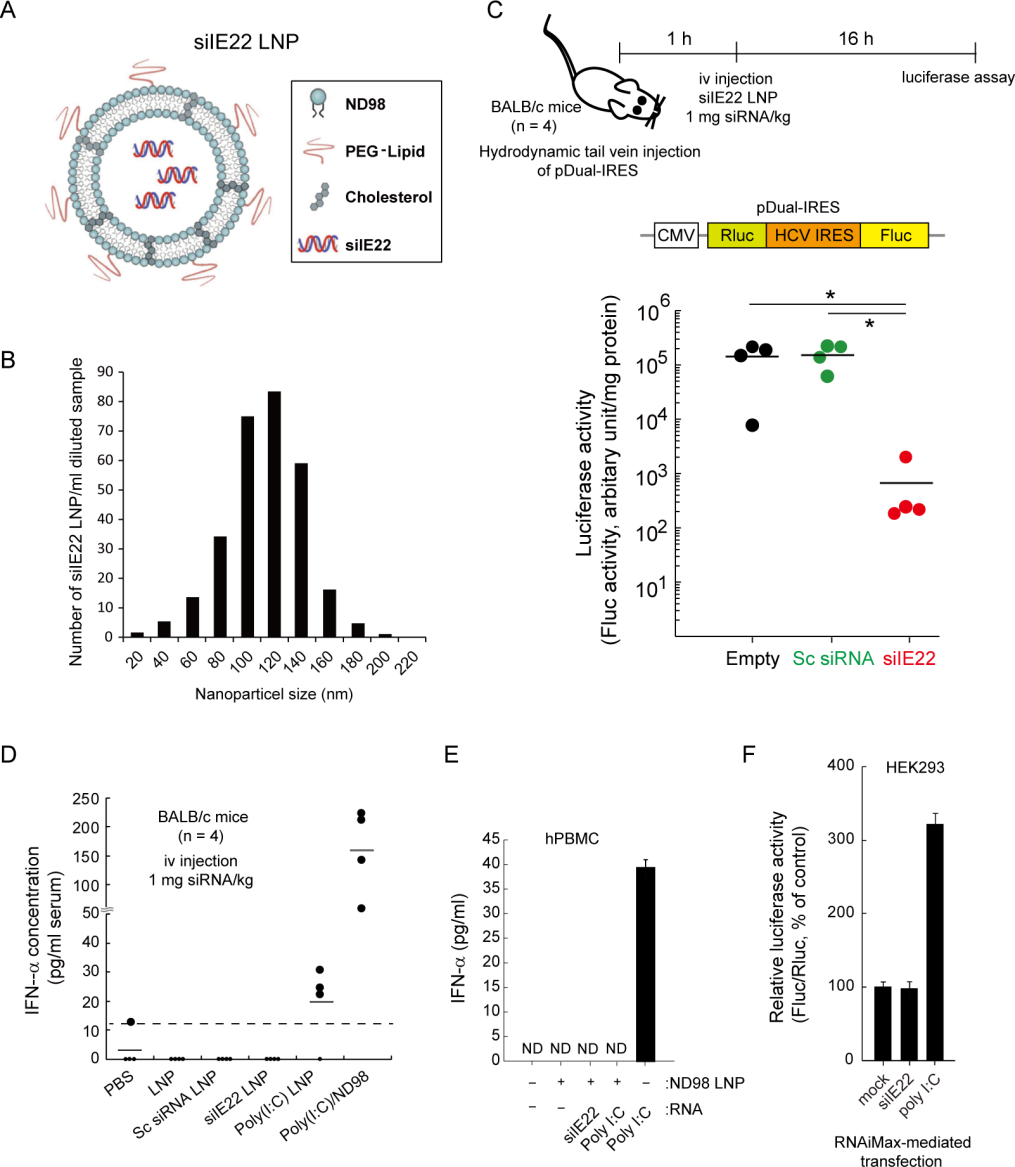
Inhibition of HCV IRES-mediated translation by systemically delivered LNP-formulated siIE22. (A and B) Schematic diagram of siIE22 LNP (A) and LNP particle size analysis (B). (C) Experimental schedule and schematic representation of the pDual-IRES plasmid. The pDual-IRES plasmid was hydrodynamically injected through the tail vein of BALB/c mice (n = 4 per group). After 1 h, mice were iv injected with siIE22 LNP at a dose of 1 mg/kg body weight. The Fluc expression level in the liver was determined 16 h after the injection. Luciferase activity is reported as RLU per mg protein. *, *P <* 0.01. (D) BALB/c mice (n = 4 per group) were iv injected with indicated siRNAs (1 mg/kg body weight) complexed with ND98 or formulated with LNP. Poly(I:C) (1 mg/kg) complexed with ND98, formulated with LNP, or free form (each in 170 μl) was administered. PBS or LNP vesicles alone were used as control treatments. Two hours later, serum IFN-α levels were quantified by ELISA. The dotted line indicates the detection limit of the assay (15 pg/ml). (E) hPMBCs grown in 96-well plates were transfected with indicated siRNA at 10 nM concentration or with 1 μg/well poly(I:C) using the lipidoid ND98 or stimulated by a direct addition of 50 μg/ml poly(I:C) to the medium. After 16 h, cell culture supernatants from stimulated cells were analyzed for IFN-α by ELISA. Data shown are from one of the two independent experiments with similar results. ND, non-detectable. (F) HEK293 cells were transfected with the luciferase expressing plasmids (IFNβ-pGL3 and pRL-TK) for the IFN-β promoter activity assay. After 6 h, cells were transfected with 100 nM siIE22 or scrambled (Sc) siRNA, or 1 μg/ml poly(I:C). After 8 h, cells were harvested for dual luciferase assays. Fluc activity was normalized to Rluc activity from the pRL-TK plasmid. Normalized luciferase activity (Fluc/Rluc) of mock-treated cells was defined as 100. Data are presented as the mean ± SD of six measurements from two independent experiments.

Inefficient siRNA delivery to target tissues and cells has been a major obstacle to the clinical development of siRNA therapeutics. We encapsulated siIE22 using a lipidoid nanoparticle formulation composed of the lipidoid ND98, cholesterol, and a PEG-lipid to facilitate the delivery of siRNA to the liver (Fig 4A). The resulting LNPs containing siIE22 (siIE22 LNPs) had a mean diameter of ~100 nm (Fig 4B), as assessed by the nanoparticle tracking analysis. In mice that were hydrodynamically injected with the pDual-IRES plasmid, the siIE22 LNP intravenously injected at a dose of 1 mg siRNA/kg body weight resulted in a significant reduction in the normalized luciferase activity by up to ~2 log_10_ (*P* < 0.01), confirming the capacity of LNPs to provide systemic delivery of siRNA (Fig 4C).

The cytosol pattern recognition receptor (PRR), retinoic acid-inducible gene 1 (RIG-I), recognizes cytoplasmic foreign RNAs including a short duplex siRNA with certain types of sequences, and induces IFN-β production [44]. We observed that siIE22 LNPs, when injected through the tail vein, did not increase IFN-α production in mice, whereas synthetic dsRNA poly(I:C), a potent activator of type-I IFN responses [44], increased serum IFN-α levels when it was delivered using ND98 or LNPs (Fig 4D). We found that poly(I:C) induced weaker IFN- α production when it was LNP-formulated compared to the IFN-α induction levels observed when poly(I:C) was administered with ND98. This finding suggests that LNP formulation may reduce the innate immune response-activating activity of siRNAs by influencing their interaction with PRRs. Furthermore, we observed little induction of IFN-α production by siIE22 LNPs in hPBMCs, while poly(I:C), known to be recognized by TLR3, induced IFN-α production when it was added directly to the culture medium, as expected (Fig 4E). Consistent with the results obtained in mice, poly(I:C), which is also recognized by the cytosolic PRRs RIG-I and MDA5 [44–46], however, did not activate IFN-α production in hPBMCs when it was delivered within LNPs. Furthermore, no intrinsic induction of the type I IFN response by siIE22 *per se* was observed in HEK293 cells by using a luciferase reporter plasmid carrying the binding site of IRF3, a transcription factor activated by the RIG-I or TLR3 pathway [38]. As shown in Fig 4F, siIE22 did not trigger promoter activation, whereas poly(I:C) that was delivered intracellularly by RNAiMax-mediated transfection increased the promoter activity by about 3-fold, as expected. In summary, these results clearly demonstrate that the RNAi potency of siIE22 is not mediated by IFN responses.

### Anti-HCV efficacy of chemically modified siIE22 derivatives

**Fig 5:**
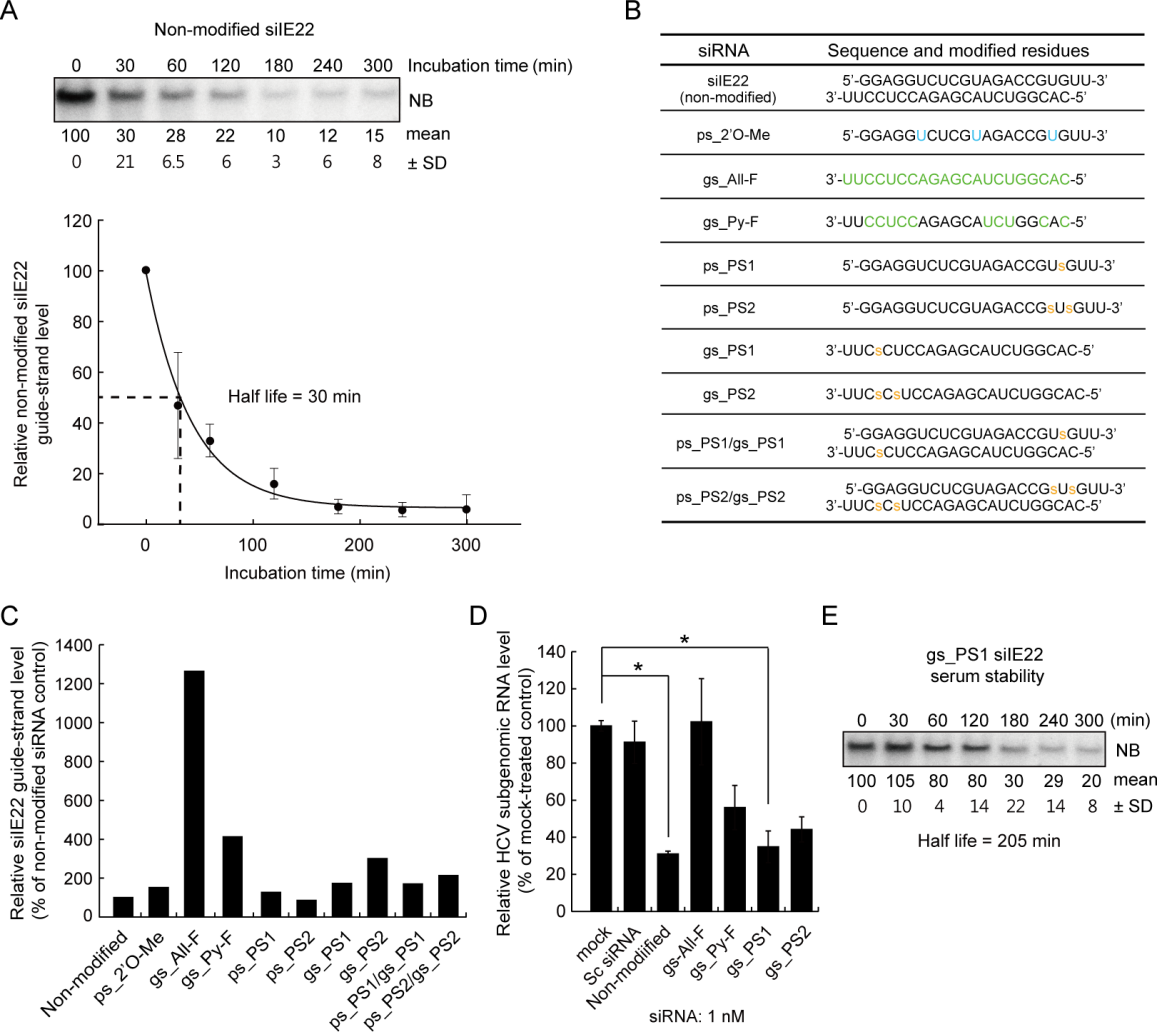
Anti-HCV efficacy of chemically modified siIE22 derivatives. (A) Non-modified siIE22 (2 μM) was incubated in 45% human plasma for the indicated time periods. RNA extracted from each sample was resolved by electrophoresis on a denaturing 15% polyacrylamide gel and subjected to northern blotting (NB) analysis for detection of siIE22 guide-strand. The Phosphorimager image shown is from one representative experiment of three independent experiments with similar results. Densitometric analysis of siIE22 guide strand signal was done using a Phosphorimager. Relative intensity of signals was plotted using SigmaPlot to estimate siIE22 guide strand half-life. Relative signal (% of signal at time 0) is shown below a representative blot. (B) Sequences of modified guide and passenger strands of siIE22 derivatives used in this study. Modified residues are shown in green or blue. “s”, phosphorothioate linkage. (C) Plasma stability of a set of the selected siIE22 derivatives was evaluated as in (A). (D) Anti-HCV activity of the selected modified siIE22 (1 nM) was evaluated in R-1 cells as in Fig 1D. *, *P <* 0.01. (E) Analysis of half-life of the gs_PS1 siIE22 as in (A).

Stabilization of synthetic siRNAs is often regarded as a prerequisite for successful *in vivo* applications. Northern blot analysis showed that the half-life of unmodified siIE22 in human plasma was ~30 min (Fig 5A). We thus chemically modified siIE22 to improve its stability against nucleases. Of several siIE22 derivatives formed with a set of passenger and guide strands of siIE22 with 2′O-Me or 2′F nucleotide modifications, or PS linkages (Fig 5B), we could select four siRNAs that showed improved stability following minimal structural modifications. Those siRNA include gs_All-F and gs_Py-F, as well as gs_PS1 and gs_PS2 siRNAs carrying one and two PS linkages on the guide strand, respectively (Fig 5C). Modified siRNAs with a PS linkage on the passenger strand were excluded because their influence on the stability was significantly lower than the effect of PS modification(s) on the guide strand. When PS-modified passenger strands were duplexed with the similarly modified corresponding guide strands, the stability of the resulting duplexes was not improved (see ps_PS1/gs_PS1 and ps_PS2/gs_PS2 in Fig 5C).

The results of antiviral efficacy test for the four selected siRNAs revealed that although gs_All-F and gs_Py-F siRNAs were more stable, they displayed reduced anti-HCV activity (Fig 5D). Based on the antiviral activity and siRNA stability, we selected gs_PS1 siIE22, which had an increased half-life of 205 min in plasma (Fig 5E) and retained anti-HCV activity of the original siRNA upon a chemical modification, for subsequent *in vivo* experiments.

### Anti-HCV efficacy of gs_PS1 siIE22 LNPs in a mouse model of HCV replication

**Fig 6:**
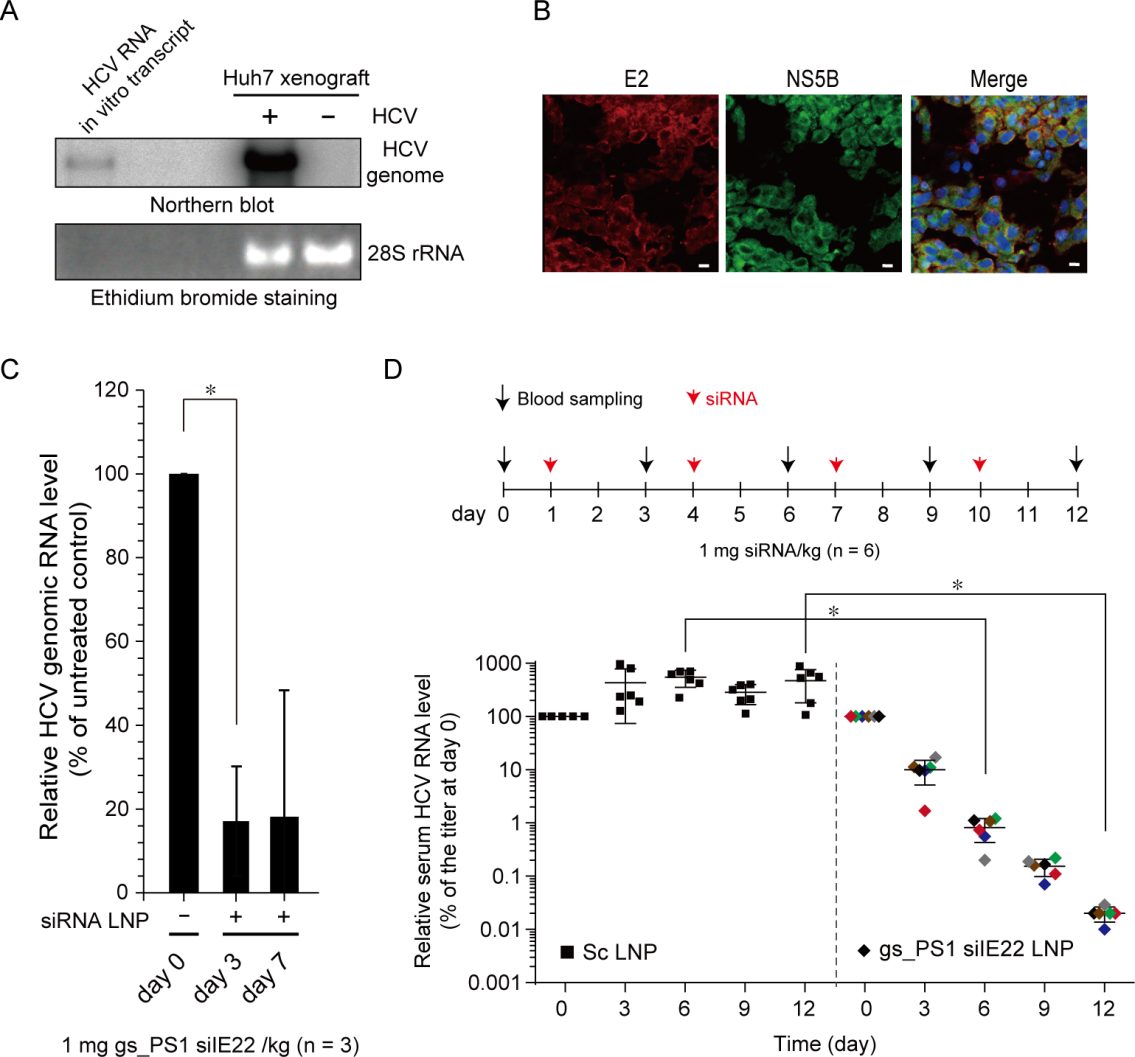
Anti-HCV efficacy of gs_PS1 siIE22 LNP in a mouse model for HCV replication. (A) HCV genome in the HCV-replicating Huh7 (+ HCV) or Huh7 (- HCV) sc xenograft was detected by northern blotting. An *in vitro* transcribed HCV RNA genome was used as a size marker. The 28S rRNA detected by ethidium bromide staining is shown as a loading control. (B) Immunostaining for HCV viral proteins (E2 and NS5B) in the xenograft at 4 weeks post-xenografting. DAPI, nuclear staining. Scale bar, 10 μm. (C) The NOD-SCID mice (n = 3) carrying HCV-replicating Huh7 xenograft were treated with gs_PS1 siIE22 LNP at a dose of 1 mg/kg body weight via tail vein injection. Shown are relative serum HCV RNA titers at the indicated time points. *, *P* < 0.01. (D) Relative serum HCV RNA titers in the mice treated with LNP-formulated Sc siRNA (Sc LNP) or gs_PS1 siIE22 LNP (1 mg/kg) once in every three days for 4 times. The data were generated from two independent experiments with a total of 6 mice per group. Each differently colored diamond represents an individual mouse. *, *P* < 0.01.

*In vivo* anti-HCV activity of siIE22 LNPs was assessed in a mouse model of HCV replication in which Huh7 cells transfected with HCV (JFH1) RNA by electroporation were implanted subcutaneously into flanks of NOD-SCID mice. In xenograft tissues retrieved from the mice at 4 weeks post-xenografting, HCV genome (Fig 6A) and viral proteins (NS5B and E2) (Fig 6B) were detected by northern blotting and immunohistochemical analysis, respectively, indicating a productive replication of HCV in the xenografts. The mice were given LNP-formulated gs_PS1 siIE22 by a single iv injection of 1 mg siRNA/kg body weight. Systemic delivery of siRNA LNPs led to more than 80% reduction in the serum HCV titer on days 3 and 7 demonstrating that sustained antiviral activity lasted for at least 7 days (Fig 6C). Administration of gs_PS1 siIE22 LNPs at a dose of 1 mg/kg did not increase alanine aminotransferase activity (data not shown), which indicated an absence of acute liver toxicity. Furthermore, when siRNA LNPs were administered once every 3 days at a dose of 1 mg/kg for a total of 4 injections, serum HCV RNA titer was reduced by more than 4 log_10_ (Fig 6D), while LNP-formulated Sc siRNA had little effect. Sequence analysis of 16 independent clones for the region targeted by siIE22 revealed that no mutations that might confer resistance to the siRNA occurred during the course of siRNA treatment (S1 Fig). This observation suggest that the target sequence has a high genetic barrier to the development of resistance.

## Discussion

Sequence-based prediction of siRNA-accessible targets in HCV IRES is challenging because of its complicated RNA structure and association with multiple host proteins [8, 20, 21, 47, 48], which can even dynamically change the structure of this *cis*-acting RNA element required for viral translation [23]. In this study, by screening a set of siRNAs tiled over the stem-loop structures III and IV of HCV IRES, we identified siIE22, an siRNA molecule that accesses the 19-nts sequence array from nt 319 to nt 337 within the IRES subdomain IIIf and displays potent anti-HCV activity. We also demonstrated that systemic administration of lipidoid nanoparticle-formulated gs_PS1 siIE22 (once every three days for a total of four injections at a dose of 1 mg/kg) resulted in a more than 4 log_10_ reduction in serum HCV RNA titer in a mouse xenograft model for HCV replication without generation of resistant variants.

Our siRNA-tiling experiments enabled us to fine map the region ranging from nt 313 to nt 343 as a site that can be accessed by the activated RISC in the cellular environment of various host components interacting with HCV IRES. We found that among multiple siRNAs tiled over IRES stem-loop structures III and IV spanning nucleotides (nts) 277–343, only a limited number of siRNAs displayed anti-HCV activity with different degrees of inhibition, depending on their target sites within pseudoknot structures. Interestingly, the mapped region locating within the pseudoknot 1 could not be accessed even *in vitro* by several 20-mer DNA antisense oligonucleotides whose binding sites overlap with the targets of siRNAs used in this study (siIE2, siIE14, and siIE23) [49]. Although interpretation of the data from a DNA microarray-based assays has to consider the stearic hindrance effects because labeled RNA probe was applied onto a chemically modified solid support carrying the immobilized DNA oligonucleotides, those previous data suggested that HCV IRES pseudoknot structures are barely accessible by oligonucleotides *per se*. In dramatic contrast, siRNA guide-strand complexed with hAgo2 appears to access the two pseudoknots in HCV IRES, as we demonstrated that several pseudoknot 1-targeting (siIE21, siIE21.5, siiE22, siIE22.5, and siIE23) and pseudoknot 2-targeting (siIE3 and siIE6) siRNAs were capable of cleaving the target RNA probe with similar potencies (Fig 2B and 2D). Despite their indistinguishable cleavage activity *in vitro*, those siRNAs showed different degrees of antiviral activity. As shown in Fig 1C, shifting the target along the conserved IRES region had a substantial impact on RNAi in the cellular context. To our surprise, the inhibitory efficacy of the highly potent, previously unidentified siRNA siIE22 was markedly affected even by a single nucleotide shift of its target site: 1-nt or 2-nt shift toward either 5′- or 3′-direction significantly reduced its antiviral potency (Fig 1C and 1D). It is worth noting, however, that those target-shifted siRNAs showed similar RNAi efficiency *in vitro* (Fig 2A and 2B) regardless of the nature of sequence at position 10 of the guide strand, where A or U were previously proposed to be favored for Ago2-mediated substrate cleavage [50]. Our results showed that the accessibility to the target, in addition to siRNA sequence and thermodynamic asymmetry of siRNA ends that would affect guide strand Ago2 loading, account for potent antiviral activity of siIE22. Thus, systematic screening of siRNAs tiled over a target site can be an effective approach for identifying potent selective siRNAs, particularly when the target interacts with multiple cellular proteins and/or other RNA segments that can hinder the access of the RISC complex. This method would complement conventional bioinformatics-assisted siRNA target prediction approaches.

Several lipid-based transfection reagents are being used *in vitro* and *in vivo* to enhance siRNA delivery in mammalian cells. Despite recent progress, effective delivery of siRNA therapeutics remains the key hurdle in siRNA therapy development [51, 52]. Nevertheless, successful systemic delivery of antiviral siRNAs has been achieved in mice and in clinical studies. Chandra et al. [53] used a cationic lipid-based nanoparticle to deliver two different HCV IRES-targeting siRNAs, which reduced the HCV RNA titer by approximately 2 log_10_ after six intravenous injections of 5 mg siRNA/kg body weight. Although the antiviral potency cannot be directly compared to that of gs_PS1 siIE22 because different siRNA delivery reagents and mouse models were used, LNP-formulated gs_PS1 siIE22 yielded a greater efficacy (more than 4 log_10_ reduction) following four injections of 1 mg siRNA/kg body weight. The siRNA delivery reagent LNP, which was formulated with the lipidoid ND98, cholesterol, and polyethylene glycol (PEG) 2000-ceramide C16, successfully delivered siRNA into the liver. As a result, a significant reduction of HCV IRES-directed reporter gene expression was observed in the liver of mice receiving siIE22 LNPs (Fig 4C). Very recently, it was reported that ARC-520, a cholesterol-conjugated siRNA formulated with Arrowhead’s proprietary Dynamic Polyconjugate, showed an antiviral effect in a phase IIa clinical trial in chronic hepatitis B virus patients [54, 55]. Currently, the phase I clinical trial for TKM-Ebola, an anti-Ebola virus siRNA formulated with the Tekmira lipid nanoparticle technology is underway [56]. Despite a considerable skepticism as to whether siRNA therapy can be effectively translated into clinical practice, antiviral siRNAs have re-engendered much promise of their clinical translation as discussed above. In preclinical studies with gs_PS1 siIE22 LNPs, we observed no noticeable adverse side-effects in mice and beagle dogs that received single injection or multiple injections (three iv injections per week and five iv injections per 2 weeks) of gs_PS1 siIE22 at doses as high as 7.69 and 6.15 mg/kg, respectively (detailed information will be reported elsewhere). This suggests that siRNA LNPs are tolerable at least in non-primate animals used in safety and toxicology studies. Furthermore, siIE22 LNPs did not induce IFN-α production in mice and in hPBMCs at all doses tested. It should be further evaluated whether siIE22 LNP therapy is suitable for treatment of HCV infection without long-term adverse effects that might be induced by siRNA off-target actions and/or its off-target tissue delivery.

Several recently approved HCV DAAs have shown outstanding clinical outcomes in chronic HCV patients [2, 3]. However, challenges such as rapid generation of resistant variants and differential SVR for different HCV genotypes remain to be overcome. Given the fact that the error-prone nature of the RdRp is responsible for the rapid emergence of resistant mutants, the potent HCV IRES-targeting siRNAs identified in the present work also may have to overcome limitations of DAAs. The siIE22 target (nts 319–337) is highly conserved across all HCV genotypes (S1 Table). Among a total 468 HCV sequences available at the HCV database web server (http://hcv.lanl.gov/content/index), we observed a single nucleotide change only in five clones, in which two clones with a C to U change can still make a G:U wobble base-pairing with the siIE22 guide strand. Thus, siIE22 LNP is a potential pan-genotypic anti-HCV agent with a high genetic selection barrier. Because of its high sequence conservation across all HCV genotype, mutations in siIE22 target are likely to be accompanied with a complete loss of or significantly lower viral infectivity due to induction of defects in viral genome translation and/or replication. Indeed, the target sequence of siIE22 remained unchanged when analyzed at day 12 after the first treatment with siRNA LNPs, while four out of sixteen sequence reads had a deletion or a U to C base substitution at the SL IIIa, IIIb, and IIIc regions (S1 Fig). Potent anti-HCV siRNAs (siIE19, siIE21, siIE21.5, siIE22, siIE22.5, and siIE23) discovered in our study can be potentially combined to further impede a potential occurrence of resistant variants and achieve longer lasting SVR with an overall improvement of treatment potency. Anti-HCV siRNAs identified in this work by siRNA tiling approaches might be beneficial for HCV patients who are not responding to current oral DAA regimens and become carriers of DAA-resistant variants.

## Supporting Information

S1 Fig Target sequence analysis of the resistance after treatment with gs_PS1 siIE22 LNP. Total RNA was extracted from the xenograft implanted subcutaneously in the NOD-SCID mouse model for HCV replication 2 days after the 4th injection of siRNA LNP (see Fig 6D experimental schedule). HCV 5′-region spanning nts 130–487 was amplified by RT-PCR. The resulting PCR products were cloned and a total of 16 independent clones were analyzed by sequencing. Among 17 individual clones analyzed, 5 clones showed sequence variations including a single G deletion at nt-189 and a T to C conversion at nts 159, 163, 211, and 233, while the siIE22 target sequence remained unchanged. WT, genotype 2a HCV (JFH1).
(TIF)

S1 Table Multiple sequence alignment of the HCV IRES region spanning nts 301–341. (PDF)
