# Supplementary figures and images for: Inhibition of Hepatitis C Virus in Mice by a Small Interfering RNA Targeting a Highly Conserved Sequence in Viral IRES Pseudoknot
Source: PLoS One. 2016 Jan 11;11(1):e0146710. doi: 10.1371/journal.pone.0146710 (PMC4713436; doi:10.1371/journal.pone.0146710)

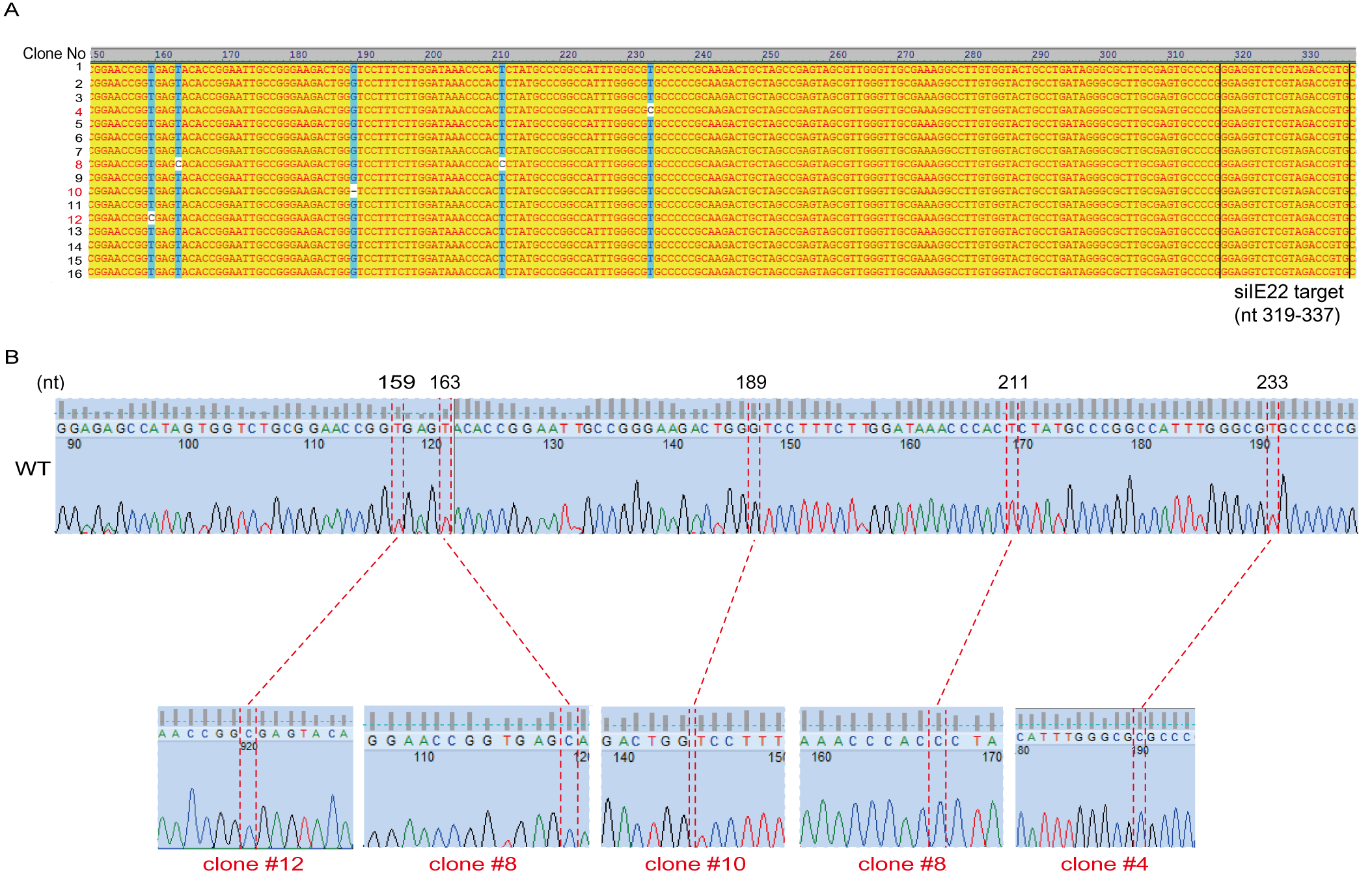

Supplement: S1 Fig — Total RNA was extracted from the xenograft implanted subcutaneously in the NOD-SCID mouse model for HCV replication 2 days after the 4th injection of siRNA LNP (see Fig 6D experimental schedule). HCV 5′-region spanning nts 130–487 was amplified by RT-PCR. The resulting PCR products were cloned and a total of 16 independent clones were analyzed by sequencing. Among 17 individual clones analyzed, 5 clones showed sequence variations including a single G deletion at nt-189 and a T to C conversion at nts 159, 163, 211, and 233, while the siIE22 target sequence remained unchanged. WT, genotype 2a HCV (JFH1). (TIF) [file pone.0146710.s001.tif]
