# Supplementary material for: Inhibition of Hepatitis C Virus in Mice by a Small Interfering RNA Targeting a Highly Conserved Sequence in Viral IRES Pseudoknot
Source: PLoS One. 2016 Jan 11;11(1):e0146710. doi: 10.1371/journal.pone.0146710 (PMC4713436; doi:10.1371/journal.pone.0146710)
Supplement: S1 Table — (PDF) [file pone.0146710.s002.pdf]

S1 Table. Multiple sequence alignment of the HCV IRES region spanning nts 301–341

Genotype	Name	Sequence [a]	GenBank No	Clone No
1a	H77	GUGCUUGCGAGUGCCCCGGGAGGUCUCGUAGACCGUGCACC	NC_004102	139
	US.BID-V182	GUGCUUGCGAGUGCCCCGGGAGGUCUCGUAGACCGUGCAAC	EU234064	2
	HEC278830	GUGCUUGCGAGUGCCCCGGGAGGUCUCGUAGACCGUGCAUC	AJ278830	1
	US.BID-V411	CGUGCACCGAGUGCCCCGGGAGGUCUCGUAGACCGUGCACC	EU155311	1
1a/2a	WO0075338-9	GUGCUUGCGAGUGCCCCGGGAGGUCUCGUAGACCGUGCACC	AX057094	1
1b/2k	RU.N687	GUGCUUGCGAGUGCCCCGGGAGGUCUCGUAGACCGUGCAUC	AY587845	1
1b	Con1	GUGCUUGCGAGUGCCCCGGGAGGUCUCGUAGACCGUGCACC	AJ238799	183
	CN.AY587016	GUGCUUGCGAGUGCCCCGGGAGGUCUCGUAGACCGUGCAUC	AY587016	38
	JP.MD6-675	GUGCUUGCGAGUGCCCCGGGAGGUCUCGUAGACCGUGCAAC	AF165056	1
	US.BID-V341	GCGCUUGCGAGUGCCCCGGGAGGUCUCGUAGACCGUGCACC	EU155300	1
	MD27	GUGCUUGCGAGUGCCCCGGGAGGUCUCGUAGAUCGUGCAUC	AF207768	1
	JP.MD9-1012	GUGCUUGCGAGAGCCCCGGGAGGUCUCGUAGACCGUGCAUC	AF165062	1
	MD26	GUGCUUGCGAGGGCCCCGGGAGGUCUCGUAGAUCGUGCACC	AF207767	1
1c	IN.Khajal	GUGCUUGCGAGUGCCCCGGGAGGUCUCGUAGACCGUGCACC	AY651061	3
1	GQ.AJ851228	GUGCUUGCGAGUGCCCCGGGAGGUCUCGUAGACCGUGCACC	AJ851228	1
2a	JP.AY746460	GUGCUUGCGAGUGCCCCGGGAGGUCUCGUAGACCGUGCACC	AY746460	9
	G2AK1	GUGCUUGCGAGUGCCCCGGGAGGUCUCGUAGACCGUGCAUC	AF169003	4
	JFH-1	GCGCUUGCGAGUGCCCCGGGAGGUCUCGUAGACCGUGCACC	AB047639	1
2b	JP.MD2b1-2	GUGCUUGCGAGUGCCCCGGGAGGUCUCGUAGACCGUGCAUC	AY232731	12
	JPUT971017	GUGCUUGCGAGUGCCCCGGGAGGCCUCGUAGACCGUGCACC	AB030907	1
2c	BEBE1	GUGCUUGCGAGUGCCCCGGGAGGUCUCGUAGACCGUGCAUC	D50409	1
2k	MD.VAT96	GUGCUUGCGAGUGCCCCGGGAGGUCUCGUAGACCGUGCACC	AB031663	1
3a	DE.HCVCENS1	GUGCUUGCGAGUGCCCCGGGAGGUCUCGUAGACCGUGCAAC	X76918	3
	K3A	GCGCUUGCGAGUGCCCCGGGAGGUCUCGUAGACCGUGUAAC	D28917	1
3b	JP.HCV-Tr	GUGCUUGCGAGUGCCCCGGGAGGUCUCGUAGACCGUGCAAC	D49374	2
3k	ID.JK049	GUGCUUGCGAGUGCCCCGGGAGGUCUCGUAGACCGUGCAUC	D63821	1
4a	EG.ED43	GUGCUUGCGAGUGCCCCGGGAGGUCUCGUAGACCGUGCACC	Y11604	9
4d	03-18	GUGCUUGCGAGUGCCCCGGGAGGUCUCGUAGACCGUGCACC	DQ418786	2
5a	GB.EUH1480	GUGCUUGCGAGUGCCCCGGGAGGUCUCGUAGACCGUGCACC	Y13184	2
	ZA.SA13	GUGCUUGCGAGUGCCCCGGGAGGUCUCGUAGACCGUGCAAC	AF064490	1
6a	HK.6a35	GUGCUUGCGAGUGCCCCGGGAGGUCUCGUAGACCGUGCAUC	DQ480513	13
	HK.6a33	GUGCUUGCGAGUGCCCCGGGAGGUCUCGUAGACCCUGCAUC	AY859526	1
	HK.6a74	GUGCUUGCGAGUGCCCCGGGAGGUCUCGUAGACGGUGCAUC	DQ480524	1
6b	Th580	GUGCUUGCGAGUGCCCCGGGAGGUCUCGUAGACCGUGCAAC	NC_009827	1
6c	TH.Th846	GUGCUUGCGAGUGCCCCGGGAGGUCUCGUAGACCGUGCAUC	EF424629	1
6d	VN.VN235	GUGCUUGCGAGUGCCCCGGGAGGUCUCGUAGACCGUGCAUC	D84263	1
6e	CN.GX004	GUGCUUGCGAGUGCCCCGGGAGGUCUCGUAGACCGUGCAUC	DQ314805	1
6f	TH.C-0044	GUGCUUGCGAGUGCCCCGGGAGGUCUCGUAGACCGUGCAUC	DQ835760	2
6g	HK.HK6554	GUGCUUGCGAGUGCCCCGGGAGGUCUCGUAGACCGUGCAUC	DQ314806	2
6h	VN.VN004	GUGCUUGCGAGUGCCCCGGGAGGUCUCGUAGACCGUGCAUC	D84265	1
6i	TH.C-0159	GUGCUUGCGAGUGCCCCGGGAGGUCUCGUAGACCGUGCAUC	DQ835762	1
	TH.Th602	GUGCUUGCGAGUGCCCCGGGAGGUCUCGUAGACCGUGCAYC	DQ835770	1
6j	TH.C-0667	GUGCUUGCGAGUGCCCCGGGAGGUCUCGUAGACCGUGCAUC	DQ835761	2
6k	CN.KM41	GUGCUUGCGAGUGCCCCGGGAGGUCUCGUAGACCGUGCACC	DQ278893	1
	CN.KM45	GUGCUUGCAAGUGCCCCGGGAGGUCUCGUAGACCGUGCACC	DQ278891	1
	VN.VN405	GUGCUUGCGAGUGCCCCGGGAGGUCUCGUAGACCGUGCAAC	D84264	1
6l	US.537796	GUGCUUGCGAGUGCCCCGGGAGGUCUCGUAGACCGUGCAUC	EF424628	1
6m	TH.B4/92	GUGCUUGCGAGUGCCCCGGGAGGUCUCGUAGACCGUGCACC	DQ835767	2
	TH.C-0185	GUGCUUGCGAGUGCCCCGGGAGGUCUCGUAGACCGUGCAAC	DQ835765	1
	TH.C-0192	GUGCUUGCGAGUGCCCCGGGAGGUCUCGUAGACCGUGCAUC	DQ835766	1
6n	CN.KM42	GUGCUUGCGAGUGCCCCGGGAGGUCUCGUAGACCGUGCAAC	AY878652	2
6o	CA.QC227	GUGCUUGCGAGUGCCCCGGGAGGUCUCGUAGACCGUGCAAC	EF424627	1
6p	CA.QC216	GUGCUUGCGAGUGCCCCGGGAGGUCUCGUAGACCGUGCAUC	EF424626	1
6q	CA.QC99	GUGCUUGCGAGUGCCCCGGGAGGUCUCGUAGACCGUGCAAC	EF424625	1
6	CN.GZ52557	GUGCUUGCGAGUGCCCCGGGAGGUCUCGUAGACCGUGCAUC	DQ278892	1
7	CA.QC69	GUGCUUGCGAGUGCCCCGGGAGGUCUCGUAGACCGUGCACC	EF108306	1

[a]A total of 468 HCV sequences available on the HCV database web server (http://hcv.lanl.gov/content/index) operated by Los Alamos National Security were analyzed to align the IRES region, spanning nts 301–341, by using ALIGN0 and QuickAlign v1programs provided by the server. Five clones representing 1.02% of aligned sequences showed a single nucleotide change, with two clones harboring a C to U change that can still make a G:U wobble base-pairing with siIE22 guide strand. The siIE22 target is shown in blue and the residues not conserved among the aligned sequences are shown in red.
